# Activity of ceftolozane-tazobactam against *Escherichia coli* isolates from U.S. veterans (2011) in relation to co-resistance and sequence type 131 (ST131) *H*30 and *H*30Rx status

**DOI:** 10.1371/journal.pone.0200442

**Published:** 2018-07-10

**Authors:** Brian D. Johnston, Paul Thuras, James R. Johnson

**Affiliations:** 1 VA Medical Center, Minneapolis, MN, United States of America; 2 Department of Medicine, Division of Infectious Diseases and International Medicine, University of Minnesota, Minneapolis, MN, United States of America; 3 Department of Psychiatry, University of Minnesota, Minneapolis, MN, United States of America; Emory University School of Medicine, UNITED STATES

## Abstract

**Background:**

*Escherichia coli* sequence type 131 (ST131), with its multidrug-resistance-associated *H*30R1 and *H*30Rx clonal subsets, causes most antimicrobial-resistant *E*. *coli* infections in the U.S., especially among veterans. The activity of ceftolozane-tazobactam (C/T), a new beta-lactamase inhibitor agent, against ST131 strains, and *E*. *coli* isolates from veterans, is undefined.

**Methods:**

We determined broth microdilution MICs for C/T and five comparators–piperacillin-tazobactam (TZP) levofloxacin (LVX), gentamicin (GEN), ceftazidime (CAZ), and meropenem (MEM)–for 595 clinical *E*. *coli* isolates, collected in 2011 from 24 Veterans Affairs Medical Centers across the U.S. Categorical resistance and MICs were compared statistically with resistance category (fluoroquinolone-susceptible, fluoroquinolone-resistant, and extended-spectrum beta-lactamase [ESBL]-producing) and with PCR-defined ST131, *H*30R1, and *H*30Rx status.

**Results:**

Resistance prevalence was ≤ 6% for C/T (6%) and MEM (0%), vs. from 8.0% (TZP) to 59% (LVX) for the other comparators. MICs generally increased by resistance category, from fluoroquinolone-susceptible through fluoroquinolone-resistant to ESBL, and by clonal subgroup, from non-ST131-*H*30 through *H*30R1 to *H*30Rx. For each comparator agent except MEM, although a significantly greater fraction of resistant than susceptible isolates were C/T-resistant, only a minority of comparator-resistant isolates were C/T-resistant (i.e., 9% if LEV-resistant, 12% if GEN-resistant, 21% if CAZ-resistant, 38% if TZP-resistant).

**Conclusions:**

C/T was broadly active against *E*. *coli* clinical isolates from veterans, notwithstanding significant variation by resistance category and ST131-*H*30R1/*H*30Rx status, outperforming all non-carbapenem comparators. C/T should prove useful as a carbapenem-sparing therapy for multidrug-resistant *E*. *coli* ST131 infections, including in veterans.

## Introduction

*Escherichia coli* sequence type 131 (ST131) is the single main cause of antimicrobial-resistant *E*. *coli* infections in the U.S. today, including those in veterans [1–3]. ST131 is tightly associated with fluoroquinolone resistance [1, 2], which within ST131 is restricted almost entirely to the recently expanded *H*30R subclone (as defined using core genome-based phylogenetic analyses), with its two sister clades, *H*30R1 and *H*30Rx [1–5]. Of these, *H*30R1 accounts for most ST131 isolates in the U.S. and is usually extended-spectrum beta-lactamase (ESBL)-negative, although in some regions is associated with the CTX-M-14 and CTX-M-27 ESBLs [6]. By contrast, *H*30Rx is closely associated with the CTX-M-15 ESBL [7–9]. As compared to other *E*. *coli*, ST131 isolates not only are more frequently antimicrobial-resistant but, when resistant, have higher MICs, at least with fluoroquinolones [10]. Thus, ST131, and especially its highly resistant *H*30R1 and *H*30Rx subsets, pose substantial treatment challenges.

The combination agent ceftolozane/tazobactam (C/T) combines ceftolozane, a new anti-pseudomonal cephalosporin, with tazobactam, a beta-lactamase inhibitor that extends ceftolozane's spectrum of activity to include ESBL-producing and other multidrug-resistant (MDR) pathogens [11]. Based on the positive results of phase 3 clinical trials, C/T received FDA approval for treatment of complicated urinary tract infections and complicated intra-abdominal infections [12].

In recent studies C/T was active against most *E*. *coli* clinical isolates, although these derived primarily from non-U.S. locales [13–15]. The activity of C/T specifically against ST131 strains, especially those from U.S. veterans, is unknown. Given the importance of ST131-*H*30R1 as a driver of the current MDR *E*. *coli* pandemic [16], the tendency with some antimicrobial agents for resistant ST131 strains to have higher MICs and MBCs than other resistant *E*. *coli* [10], and the prominence of ST131 among veterans [1], we sought to determine C/T susceptibility in relation to both co-resistance phenotypes and ST131 subclone status among *E*. *coli* isolates collected from 24 Veterans Affairs medical centers (VAMCs) across the U.S. in 2011.

## Methods

### Isolates

As reported elsewhere [1], representative sets of *E*. *coli* clinical isolates were collected in 2011 from 24 VA medical centers distributed widely across the U.S. Each center provided ~10 each consecutive fluoroquinolone-susceptible (FQ-S) and fluoroquinolone-resistant (FQ-R) isolates (~20 total), without regard for other resistance phenotypes (including possible cephalosporin resistance or ESBL production). Additionally, due to the comparative rarity of ESBL-producing isolates, each center also provided up to 10 such isolates from 2010–2011. These isolates were classified as ESBL-producing by the source laboratories based on each laboratory's then-current methods and criteria. In total, 595 isolates were collected (within three resistance categories: 234 FQ-S, 238 FQ-R, and 123 ESBL).

The research laboratory performed species confirmation and PCR genotyping to detect ST131 [17] and its *H*30 [1] and *H*30Rx [8] clonal subsets. Based on previous core genome analyses [1–5], ST131 isolates that tested as *H*30 and were FQ-R were considered to represent the *H*30R clade, and *H*30R clade members that tested negative for *H*30Rx were considered to represent the *H*30R1 subclone. As reported elsewhere, of the 595 isolates tested, 260 (43.7%) qualified as ST131-*H*30, 259 (43.5%) as ST131-*H*30R, and 87/259 of these (33.6%; 14.6% of 595) as *H*30Rx, leaving 179 (30% of 595) *H*30R1 isolates. Notably, a single ST131 isolate tested as *H*30 by PCR but was FQ-S, so presumably represented the ancestral *H*30S subset within ST131-*H*30 [4]; it therefore was analyzed as non-*H*30R1.

### Susceptibility testing

We newly determined broth microdilution MICs for C/T and five comparator agents, including piperacillin-tazobactam (TZP), levofloxacin (LVX), gentamicin (GEN), ceftazidime (CAZ), and meropenem (MEM), by using Clinical and Laboratory Standards Institute (CLSI) specified procedures and reference strains [18]. CLSI-specified breakpoints were used to assign categorical interpretations with intermediate and resistant considered as resistant [19]. For C/T and CAZ the initial concentrations tested were from 0.25 to 32 mg/L, in doubling dilutions. If growth was seen at 32 mg/L, additional doubling dilutions were tested, up to 256 mg/L. The dilution range for other agents as follows: TZP (2.0 to 256 mg/L), GEN (0.25 to 32 mg/L), LEV (0.125 to 16 mg/L) and MEM (0.016 to 8 mg/L).

### Statistical methods

Comparisons were tested using a chi-square test for dichotomous variables and the Mann-Whitney U test for continuous variables. P < .05 was the criterion for statistical significance.

## Results

### Categorical resistance, overall and by clonal subset

Among the 595 total study isolates, resistance prevalence values ranged by agent from 0% (MEM) to 59% (LVX); C/T exhibited the second lowest value (6%) (Table 1). For each agent except MEM, resistance prevalence tended to increase by resistance category, from FQ-S through FQ-R to ESBL, and by clonal subgroup, from non-*H*30R1 through *H*30R1 to *H*30Rx. The two exceptions to this trend that involved absolute prevalence differences of > 5% included a slightly higher prevalence (i) of LVX resistance among the FQ-R isolates (100%, by definition) than among the ESBL isolates (92%), and (ii) of GEN resistance among the *H*30R1 isolates (42%) than among the *H*30Rx isolates (32%). Nearly all resistance prevalence differences across resistance categories and clonal subgroups were statistically significant (Table 1).

**Table 1:** Prevalence of resistance to ceftolozane/tazobactam and comparator agents in relation to resistance category and *H*30 subclone status among 595 *E*. *coli* clinical isolates from veterans.

	Resistance prevalence by resistance category, no. (column %)					Resistance prevalence by clonal subgroup, no. (column %)			
Agent^a^	Total (n = 595)	FQ-S (n = 234)	FQ-R (n = 238)	ESBL (n = 123)	P^b^	non-*H*30^c^ (n = 335)	*H*30R1^c^ (n = 172)	*H*30Rx^c^ (n = 87)	P^b^
C/T	35 (6)	1 (0.4)	8 (3)	26 (21)	< 0.001	16 (5)	5 (3)	14 (16)	<0.001
TZP	47 (8)	5 (2)	20 (8)	22 (18)	< 0.001	19 (6)	8 (5)	19 (22)	< 0.001
GEN	155 (26)	15 (6)	83 (35)	57 (46)	< 0.001	54 (16)	73 (42)	28 (32)	< 0.001
LVX	351 (59)	0 (0)	238 (100)	113 (92)	< 0.001	92 (28)	172 (100)	87 (100)	< 0.001
CAZ	140 (24)	6 (3)	26 (11)	108 (88)	< 0.001	54 (16)	29 (17)	57 (66)	< 0.001

FQ-S, fluoroquinolone-susceptible, non-extended-spectrum beta-lactamase (ESBL) producing; FQ-R, fluoroquinolone-resistant (some were ESBL-producing); ESBL, extended-spectrum beta-lactamase producing (most were FQ-R); C/T, ceftolozane/tazobactam; TZP, piperacillin/tazobactam; GEN, gentamicin; LVX, levofloxacin; CAZ, ceftazidime.

^a^No resistance was detected to meropenem (MEM).

^b^P values as determined by chi-square tests for three-group comparisons.

^c^non-*H*30, not a member of the ST131-*H*30R1 or *H*30Rx subclone (includes some ST131 strains); *H*30R1, fluoroquinolone resistance-associated subclone within ST131 (excludes *H*30Rx); *H*30Rx, ESBL-associated ST131 subclone within *H*30 (excludes *H*30R1). One *H*30 isolate was fluoroquinolone-susceptible, indicating membership in the *H*30S subclone within *H*30; it was excluded from analyses involving *H*30 subclone status (which thus had total n = 594, not n = 595).

### MICs in relation to resistance category

Overall, MICs ranged from below to above the boundaries of the tested drug concentration range for each agent except GEN (minimum, 0.5 mg/L) and MEM (maximum, 1.0 mg/L) (Table 2). After MEM (MIC_50_ and MIC_90_ < 0.06 mg/L), C/T exhibited the lowest MIC_50_ and MIC_90_ values. For each agent MICs varied significantly in relation to resistance category (P < .001 for all three-group comparisons; P < .01 for all pairwise coparisons, except GEN and LEV [FQ-R vs. ESBL] and MEM [FQ-S vs. FQ-R]), usually along an increasing gradient from FQ-S through FQ-R to ESBL. For MEM (which exhibited low MICs throughout) this was evident only from the maximal MIC values, and for LVX mainly from the MIC_50_ values (Table 2).

**Table 2:** MICs for ceftolozane/tazobactam and comparator agents in relation resistance category among 595 *Escherichia coli* clinical isolates from veterans.

	MICs (mg/L)														
	Total (n = 595)			FQ-S (n = 234)			FQ-R (n = 238)			ESBL (n = 123)			P^a^		
Agent	MIC_50_	MIC_90_	Range	MIC_50_	MIC_90_	Range	MIC_50_	MIC_90_	Range	MIC_50_	MIC_90_	Range	FQ-S vs. FQ-R	FQ-S vs. ESBL	FQ-R vs. ESBL
C/T	0.5	1.0	< 0.25, > 256	0.5	0.5	< 0.25, 4.0	0.5	1.0	< 0.25, > 256	1.0	16	< 0.25, 256	< 0.001	< 0.001	< 0.001
TZP	< 2	8	< 2, > 256	< 2	4	< 2, > 256	< 2	16	< 2, > 256	4	128	< 2, 256	< 0.001	< 0.001	< 0.001
GEN	1.0	2.0	0.5, > 32	1.0	4.0	0.5, > 32	2.0	>32	0.5, > 32	2.0	> 32	0.5, > 32	< 0.001	< 0.001	0.67
LEV	16	>16	< 0.125, > 16	< 0.125	0.25	< 0.125, 1.0	16	>16	4, > 16	> 16	> 16	< 0.125, > 16	< 0.001	< 0.001	0.466
CAZ	2	128	< 0.5, > 256	1.0	2	< 0.5, 64	2	8	< 0.5, > 256	64	> 256	1.0, > 256	< 0.001	< 0.001	< 0.001
MEM	< 0.06	< 0.06	< 0.06, 1.0	< 0.06	< 0.06	< 0.060, 0.125	< 0.06	< 0.06	< 0.06, 1.0	<0.06	<0.06	< 0.060, 0.25	0.32	0.001	0.006

FQ-S, fluoroquinolone-susceptible; FQ-R, fluoroquinolone-resistant; ESBL, extended-spectrum beta-lactamase producing

FQ-S, fluoroquinolone-susceptible, non-extended-spectrum beta-lactamase (ESBL) producing; FQ-R, fluoroquinolone-resistant (some were ESBL-producing); ESBL, extended-spectrum beta-lactamase producing (most were FQ-R); C/T, ceftolozane/tazobactam; TZP, piperacillin/tazobactam; GEN, gentamicin; LVX, levofloxacin; CAZ, ceftazidime; MEM, meropenem.

^a^ P values (as determined by the Mann-Whitney U Test) for all pairwise comparison between the three resistance categories. For all three-group comparisons across resistance categories, P < 0.001 (as determined by the Kruskal-Wallis test.).

### MICs in relation to clonal subgroup

The three clonal subgroups (non-ST131-*H*30, *H*30R1, and *H*30Rx) exhibited significant MICs differences for all studied agents except MEM, including in 5 of 6 three-group comparisons and in 12 of 18 pairwise comparisons (Table 3). The rank order of subgroups for MICs varied by agent. For C/T, exceptionally, the rank order of the subgroups (by increasing MICs) was *H*30R1 < non-*H*30 < *H*30Rx, with *H*30R1 having the lowest MICs overall. For TZP, MICs of non-*H*30 and *H*30R1 isolates were similarly low, and those of *H*30Rx isolates comparatively high. By contrast, for GEN and LEV, MICs of non-*H*30 isolates were comparatively low, and those of *H*30R1 and *H*30Rx isolates similarly high. Finally, CAZ MICs exhibited an ascending gradient, from non-*H*30, through *H*30R1, to *H*30Rx.

**Table 3:** MICs for ceftolozane/tazobactam and comparator agents in relation to *H*30 subclone status among 594 *Escherichia coli* clinical isolates from veterans.

	MICs (mg/L)											
	non-ST131-*H*30^a^ (n = 335)			*ST131-H*30R1^a^ (n = 172)			*ST131-H*30Rx^a^ (n = 87)			P^b^		
Agent	MIC_50_	MIC_90_	Range	MIC_50_	MIC_90_	Range	MIC_50_	MIC_90_	Range	non-*H*30 vs *H*30R1	non-*H*30 vs *H*30Rx	*H*30R1 vs *H*30Rx
C/T	0.5	1.0	< 0.25, > 256	0.5	1.0	< 0.25, 32	1.0	8.0	< 0.25, 256	< 0.001	< 0.001	< 0.001
TZP	< 2	8.0	< 2, > 256	< 2	8.0	< 2, > 256	4.0	128	< 2, > 256	0.19	0.001	< 0.001
GEN	1.0	> 32	0.5, > 32	2.0	> 32	0.5, > 32	2.0	> 32	0.5, > 32	< 0.001	< 0.001	0.31
LEV	< 0.125	> 16	< 0.125, > 16	> 16	> 16	8, > 16	16	> 16	8.0, > 16	< 0.001	< 0.001	0.55
CAZ	1.0	32	< 0.25, > 256	2	32	1.0, > 256	64	> 256	1.0, > 256	< 0.001	< 0.001	< 0.001
MEM	< 0.06	< 0.06	< 0.06, 1.0	< 0.06	< 0.06	< 0.06, 0.125	< 0.06	< 0.06	< 0.06, 0.125	0.58	0.13	0.08

C/T, ceftolozane/tazobactam; TZP, piperacillin/tazobactam; GEN, gentamicin; LEV, levofloxacin; CAZ, ceftazidime; MEM, meropenem.

^a^The non-ST131-*H*30 group include all non-ST131 isolates plus those ST131 isolates that do not belong to the indicated ST131 subclones (*H*30R1 or *H*30Rx).

^b^P values based on Mann-Whitney U Test

### Prevalence of C/T resistance vs. resistance to comparator agents

The prevalence of co-resistance to C/T among isolates resistant to TZP, CAZ, GEN, or LEV ranged by comparator agent from 9% (LEV) to 38% (TZP), and was significantly higher among such isolates than among isolates susceptible to the particular agent (Table 4). C/T co-resistance was more frequent among isolates resistant to other beta-lactam agents (TZP, 38%; CAZ, 21%) than among those resistant to non-beta-lactam agents (GEN, 12%; LEV, 9%).

**Table 4:** Prevalence of resistance to ceftolozane/tazobactam among *Escherichia coli* clinical isolates susceptible or resistant to alternative agents.

	Proportion (%) resistant to C/T		
Alternative agent	If susceptible to alternative agent	If resistant to alternative agent	P^a^
TZP	17/548 (3)	18/47 (38)	< 0.001
GEN	16/440 (4)	19/155 (12)	< 0.001
LEV	3/244 (1.0)	32/351 (9)	< 0.001
CAZ	1/455 (0.2)	34/140 (21)	< 0.001
MEM	35/595 (6)	NA^b^	NA^b^

C/T, ceftolozane/tazobactam; TZP, piperacillin/tazobactam; GEN, gentamicin; LEV, levofloxacin; CAZ, ceftazidime; MEM, meropenem.

^a^P values as determined by chi-square tests

^b^NA, not applicable (no resistance detected to MEM).

## Discussion

Here, among 595 recent *E*. *coli* clinical isolates from U.S. veterans (2011) [1], we assessed MICs and categorical resistance for the novel combination agent C/T and 5 comparator agents, then compared these results statistically with resistance category (FQ-S, FQ-R, and ESBL) and ST131-based clonal subgroup (non-*H*30, *H*30R1, and *H*30Rx). Our findings support four main conclusions. First, C/T was broadly active, encountering resistance in only 6% of study isolates overall (despite deliberate enrichment for resistant strains), which was the lowest value among the five non-carbapenem study agents. Second, C/T MICs and categorical resistance prevalence increased significantly by resistance category, from FQ-S through FQ-R to ESBL. Third, for each comparator agent the great majority of resistant isolates remained susceptible to C/T. Fourth, although susceptibility to C/T and the comparators varied significantly in relation to clonal background, with *H*30Rx isolates being most resistant, C/T performed well even against *H*30Rx isolates (16% C/T-resistant; C/T MIC_90_ = 8.0).

Regarding the observed 6% overall resistance prevalence for C/T, even this favorable statistic–which would qualify C/T for use in empirical treatment of pyelonephritis according to Infectious Diseases Society of America guidelines [20]–doubtless overstates the actual C/T resistance prevalence among unselected clinical *E*. *coli* isolates from veterans. This is because the study population was deliberately enriched for FQ-R and ESBL isolates, which our results show are more likely to be C/T-resistant than are other *E*. *coli*.

Regarding the progressive increase in categorical resistance to C/T and C/T MICs across the three resistance categories (FQ-S, FQ-R, and ESBL), this is consistent with the known presence within the FQ-R group of an ESBL-producing subset, with some ESBLs, possibly in conjunction with other resistance mechanisms, likely conferring resistance to C/T. Despite these trends, C/T still almost always exhibited better susceptibility statistics than did the other non-carbapenem study agents, including within the ESBL isolate group (21% C/T resistance, vs. 18%-92% for the non-MEM comparator agents). The highest observed resistance prevalence for C/T, which was among TZP-resistant isolates, was only 38%, indicating that C/T could serve as an alternate agent for nearly two-thirds of TZP-resistant isolates, let alone for nearly all isolates resistant to the other non-carbapenem comparators.

Regarding the clonal distribution of resistance to C/T and the comparators, the observed patterns were consistent with class-specific resistance mechanisms that are known to be clonally associated [5, 17, 21, 22]. Specifically, for beta-lactams (C/T, TZP, and CAZ), resistance prevalence and MICs were higher among (ESBL-associated) *H*30Rx isolates than among the non-*H*30 and the *H*30R1 isolates, which did not differ greatly from one another. By contrast, for GEN and LEV, resistance prevalence and MICs were higher among the *H*30R1 and the *H*30Rx isolates (which did not differ greatly from one another) than among the non-*H*30 isolates.

An additional unexpected finding was the statistically significantly (albeit subtly) higher C/T MICs among the non-*H*30 isolates than the *H*30R1 isolates, as reflected in the upper limit of the range (Table 3). This may have been due to a larger ESBL-producing subset among the non-*H*30 isolates than among the *H*30R1 isolates, although such a difference is not apparent from the susceptibility data for TZP or CAZ. Different results could be anticipated in regions where a substantial fraction of *H*30R1 isolates produce CTX-M-14 or CTX-M-27 [6, 23].

The study is limited by its *in vitro* nature, since MICs may not correspond with clinical efficacy, its focus on veterans (who may not represent well the general U.S. population), the lack of attention to genetic resistance mechanisms, the uncertain current relevance of isolates from 2011, and the geographic restriction to the U.S. Study strengths include the broadly distributed (within the U.S.) strain collection, the comparisons with the clinically relevant ST131 clonal subgroups *H*30R1 and *H*30Rx, and the inclusion (for both MICs and categorical resistance) of a range of relevant comparator agents.

In summary, within broadly representative set of clinical *E*. *coli* isolates from U.S. veterans from 2011 we found that C/T is broadly active, albeit with significant variation in relation to co-resistance and to *H*30R1 and *H*30Rx subclone status, and outperformed all non-carabapenem comparators. Thus, C/T should prove useful as a carbapenem-sparing agent against multidrug-resistant *E*. *coli* infections in veterans, including those caused by ST131-*H*30R1 and *H*30Rx strains.

## Supporting information

S1 Study Dataset Source data, MIC results, and molecular typing results for 595 *Escherichia coli* isolates from U.S. veterans. (XLSX)
